# Role of microRNA-133a in epithelial ovarian cancer pathogenesis and progression

**DOI:** 10.3892/ol.2014.1841

**Published:** 2014-01-29

**Authors:** JIE LUO, JIANHONG ZHOU, QI CHENG, CAIYUN ZHOU, ZHIMING DING

**Affiliations:** Department of Gynecologic Oncology, Women’s Hospital, School of Medicine, Zhejiang University, Hangzhou, Zhejiang 310006, P.R. China

**Keywords:** microRNA-133a, epithelial ovarian cancer, tumor stage, metastasis, invasion, migration

## Abstract

It has been demonstrated that microRNA (miR)-133a is downregulated in a number of human malignancies and is closely associated with the progression of tumors. The present study was conducted to investigate the contribution of miR-133a to the initiation and malignant progression of human epithelial ovarian cancer (EOC). Quantitative polymerase chain reaction was employed to detect the expression of miR-133a in the human EOC OVCAR-3 cell line, normal human ovarian surface epithelial (tsT) cells and 96 tissue samples, including 70 EOC tissues and 26 normal ovarian tissue sections. Additionally, analysis of the correlation between miR-133a levels and clinicopathological characteristics was carried out. The effect of miR-133a on cell viability, apoptosis, invasion and migration was investigated following transfection with miR-133a mimics and negative control small interfering RNA in OVCAR-3 cells. Marked downregulation of miR-133a was observed in the OVCAR-3 cell line and primary tumor samples, and it was found that reduced miR-133a expression significantly correlated with advanced clinical stages, poor histological differentiation and lymph node metastasis. Furthermore, OVCAR-3 cell viability, invasion and migration were significantly inhibited, while cell apoptosis was increased, following transfection of miR-133a mimics. The present study reveals the critical role that miR-133a plays in EOC pathogenesis and development, indicating that it may act as a promising biomarker for predicting EOC progression and as a potential target for gene therapy.

## Introduction

It has been demonstrated that epithelial ovarian cancer (EOC) serves as one of the most common types of cancer in females and is the leading cause of mortality from gynecological malignancy worldwide (1). In previous years, a number of patients with EOC have benefited from refined and more radical surgical techniques, as well as adjuvant combination chemotherapy. However, the overall 5-year survival rate of ~70% of patients was low (~33%), as diagnosis was made at the advanced stages, when extensive cell invasion and migration had already taken place in the abdominopelvic cavity (2). The potential molecular mechanisms which cause the initiation, chemotherapy resistance and development of metastasis in EOC are not well understood. Therefore, it is of great importance to identify the effector molecules and/or signaling pathways responsible for EOC pathogenesis and progression, in order to optimize treatment strategies.

MicroRNAs (miRNAs) are small (20–24 nucleotides) noncoding RNA gene products that post-transcriptionally regulate gene expression by negatively modulating the stability or translational efficiency of their target mRNAs (3). Evidence for the importance of miRNAs in cancer came from the finding that miRNA genes were specifically deleted in leukemia (4). Additionally, miRNAs have been shown to be differentially expressed in a number of other cancer types (5,6). Taken together, miRNAs are considered to be the critical factors in numerous malignancies, acting as tumor suppressors or oncogenes (7,8). Previous studies have also illustrated that various miRNAs may lead to invasion and metastasis in EOC (9–13).

Among the miRNAs, miR-133a is regarded as one of the major tumor suppressor miRNAs. Aberrant miR-133a expression was previously reported in human malignancies, including bladder cancer (5), head and neck cancer (14), rhabdomyosarcoma (15), esophageal cancer (16), colon cancer (17), tongue cancer (18) and renal cell carcinoma (19), using high-throughput technology, including miRNA oligonucleotide arrays and quantitative polymerase chain reaction (qPCR). However, it remains unknown whether miR-133a has a functional role in EOC. Thus, the present study was performed to investigate the expression of miR-133a in EOC tissues and the human EOC OVCAR-3 cell line by qPCR. Additionally, the effects of miR-133a on OVCAR-3 cell proliferation, apoptosis, invasion and migration were analyzed.

## Materials and methods

### Human tissues and cell lines

A total of 96 tissue samples, including 70 epithelial ovarian cancer tissues and 26 normal ovarian tissue sections from the Affiliated Obstetrics and Gynecology Hospital, Zhejiang University School of Medicine (Hangzhou, China) were collected between January 2009 and June 2012. Additionally, ovarian tumor samples from debunking surgery and the corresponding pathological data were collected. Patients with a previous or secondary malignancy, or having previously undergone radiation therapy, chemotherapy, or immunotherapy, were excluded from the study. The histopathological diagnoses were performed according to the World Health Organization criteria (20) and the tumor histotypes included 38 serous and 32 non-serous types. All tumor stages were determined based on the International Federation of Gynecology and Obstetrics standards (FIGO) (21). The stage breakdown was as follows: n=8 for stage I, n=12 for stage II, n=35 for stage III and n=15 for stage IV. Samples from patients who had undergone oophorectomy for benign uterine pathologies were used as normal control tissues. The study was approved by the Medical Ethics Committee of the Women’s Hospital, Zhejiang University (Hangzhou, China) and all patients provided informed consent. All fresh specimens were initially stored at 4°C for 24 h in RNAlater (Ambion, Carlsbad, CA, USA) and subsequently at −80°C in liquid nitrogen until further use. The human EOC OVCAR-3 cell line and normal human ovarian surface epithelial [OSE(tsT)] cells were supplied by China Center for Type Culture Collection (Wuhan, China). Cells were cultured in Dulbecco’s modified Eagle’s medium (DMEM; Gibco-BRL, Carlsbad, CA, USA) supplemented with 10% fetal bovine serum (Biological Industries, Kibbutz Beit Haemek, Israel) and incubated at 37°C in 5% CO_2_.

### RNA extraction and qPCR

In order to perform qPCR analysis, total RNA was first extracted using miRNeasy kit (Qiagen, Hilden, Germany) following the manufacturer’s instructions. cDNA was synthesized from total RNA using miR-133a reverse transcription (RT) primer. The miR-133a RT primer was: 5′-GTCGTATCCAGTGCAGGGTCCGAGGTATTCGC ACTGGATACGACACAGCT-3′. The miR-133a PCR primers were: Forward, 5′-CTGCATTGGTCCCCTTCAAC-3′ and reverse, 5′-CAGTGCAGGGTCCGAGGTAT-3′. miRNA expression was detected using qPCR with SYBR Green RT-PCR kit (Takara Bio, Inc., Shiga, Japan). The reaction was incubated at 94°C for 4 min, followed by 35 cycles of 20 sec at 94°C, 30 sec at 60°C and 30 sec at 72°C. U6 small nuclear RNA was used as an endogenous internal standard control. The threshold cycle (Ct) was determined as the fractional cycle number at which the fluorescence passed the fixed threshold. All experiments were repeated twice and, in each experiment, samples were assayed in triplicate. Data were expressed as the expression level of miR-133a relative to that of the internal control U6, using the 2^−ΔΔCt^ method (22).

### miR-133a transfection

miR-133a mimics were obtained from Shanghai GenePharma Co., Ltd (Shanghai, China). These included synthetic small duplex sequences of miR-133a-RNA able to be bioprocessed into mature miR-133a in the cells. The negative control (NC) sequence, which was not homologous to any human genome sequence, was used to eliminate any potential non sequence-specific effects. Primers for miR-133a were as follows: Sense, 5′-UUUGGUCCCCUUCAACCAGCUG-3′ and antisense, 5′-GCUGGUUGAAGGGGACCAAAUU-3′; NC siRNA sense, 5′-UUCUCCGAACGUGUCACGUTT-3′ and antisense, 5′-ACGUGACACGUUCGGAGAATT-3′. The transfection of miR-133a mimics into cells was carried out using Lipofectamine 2000 (Invitrogen Life Technologies, Carlsbad, CA, USA). The cells were cultured using complete medium without antibiotics, and Lipofectamine 2000 and miR-133a mimics were diluted with serum-free medium.

### Analysis of cell viability in vitro

MTT assay was used to analyze the cell viability of OVCAR-3 transfected with NC or miR-133a mimics (23). Briefly, cells were seeded into 96-well plates and transfected. In the indicated time periods, 0.1 ml fresh medium containing 0.5 mg/ml MTT was used to replace an equal volume of spent medium. Following incubation at 37°C for 4 h, the medium was replaced by 0.1 ml dimethyl sulfoxide (Sigma-Aldrich, St. Louis, MO, USA) and agitated at room temperature for 10 min. The absorbance was measured by a spectrometer at a wavelength of 490 nm.

### Detection of apoptosis

OVCAR-3 cells were transfected with NC or miR-133a mimics for 48 h. Next, cell culture medium was replaced with serum-free DMEM. At the indicated time periods following serum deprivation, cells were harvested, washed, resuspended in the staining buffer and examined with the Vybrant Apoptosis Assay kit (Invitrogen Life Technologies). Stained cells were identified by FACSCalibur and data were analyzed with CellQuest software (both from BD Biosciences, Franklin Lakes, NJ, USA). Annexin V-positive and propidium iodide-negative cells were considered to be apoptotic cells.

### Invasion assay

Invasion assays were performed using transwell invasion chambers coated with Matrigel (50 μl per filter; BD Biosciences) according to the manufacturer’s instructions. OVCAR-3 cells were transfected with NC or miR-133a mimics for 48 h and transferred onto the top of Matrigel-coated invasion chambers in serum-free DMEM (1×10^5^ cells per transwell). DMEM containing 10% fetal calf serum was added to the lower chambers. Following incubation for 24 h, cells remaining on the top of the filter were removed and those that migrated to the lower surface were fixed in 90% alcohol and subjected to crystal violet staining. The number of migrated cells on the lower surface of the membrane was counted under a microscope in 10 fields with a magnification of ×400. The invasion assays were carried out in triplicate.

### Scratch migration assay

OVCAR-3 cells were transfected with NC or miR-133a mimics and grown to confluence. Subsequently, a scratch was set though the dish and cells were cultured under standard conditions for 24 h. Following several washes, plates were photographed and the cell migration was evaluated by counting cells that had migrated from the wound edge.

### Statistical analysis

Data are represented as the mean ± standard error of the mean. Statistical analysis was carried out using a Student’s t-test. All statistical analyses were performed using SPSS version 17.0 (SPSS, Inc., Chicago, IL, USA). P<0.05 was considered to indicate a statistically significant difference.

## Results

### miR-133a is downregulated in OVCAR-3 cells and primary EOC tumor samples

**Figure 1:**
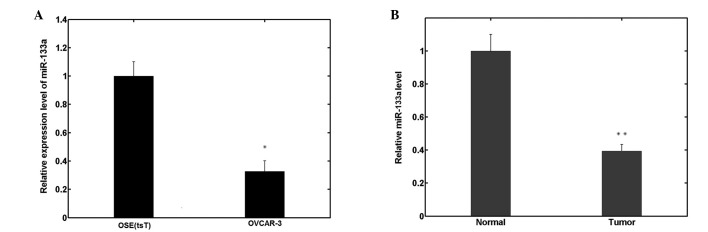
Relative miR-133a levels were measured by quantitative polymerase chain reaction in the (A) human epithelial ovarian cancer OVCAR-3 cell line and (B) primary tumor samples. miR-133a expression was significantly decreased in OVCAR-3 cells compared with OSE(tsT) cells. miR-133a expression was also significantly downregulated in primary tumors compared with normal ovarian tissues. miR, microRNA; OSE, ovarian surface epithelial. ^*^P<0.05 and ^**^P<0.01 vs. the control group.

In order to investigate the biological roles of miR-133a in human EOC pathogenesis and progression, miR-133a expression was measured by qPCR in human OSE(tsT) and OVCAR-3 EOC cell lines. As shown in Fig. 1A, miR-133a expression significantly decreased in OVCAR-3 cells compared with OSE(tsT) cells. Similarly, among the 70 ovarian cancer samples analyzed, the relative expression of miR-133a was also significantly downregulated compared with the 26 normal ovarian tissues, as shown in Fig. 1B.

### Decreased miR-133a expression is associated with clinicopathological features

Table I shows that the relative expressions of miR-133a were significantly lower in advanced stage and grade 3 tumor samples compared with early stage and grade 1 and 2 tumor samples. The same differences were observed between the lymph node-positive and -negative group. However, there were significant differences between serous and non-serous tissue samples.

### miR-133a reduces cell viability and promotes cell apoptosis

**Figure 2:**
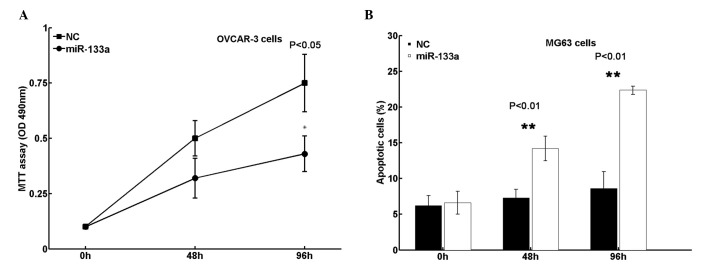
(A) MTT assay was performed to determine cell viability. Transfection of miR-133a mimics reduced cell viability in human epithelial ovarian cancer OVCAR-3 cells. (B) Cell apoptosis was analyzed using a staining buffer and apoptosis assay. Cell apoptosis is increased following restoration of miR-133a expression. miR, microRNA; NC, negative control; OD, optical density. ^*^P<0.05 and ^**^P<0.01 vs. the control group.

In order to determine whether miR-133a functions as a tumor suppressor in EOC, cell viability and apoptosis were analyzed in the present study. It was revealed that transfection of miR-133a mimics significantly reduced cell viability in OVCAR-3 cells (Fig. 2A). Additionally, cell apoptosis in OVCAR-3 cells increased following restoration of miR-133a expression (Fig. 2B). Collectively, these results indicate that miR-133a suppresses EOC growth *in vitro*.

### miR-133a affects cell invasion and migration in vitro

**Figure 3:**
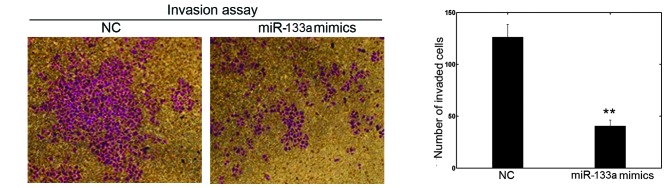
An invasion assay was performed to determine the effect of miR-133a on cell invasion (magnification, ×400). Following transfection with miR-133a mimics or NC in OVCAR-3 cells, a significant downregulation of invasion into Matrigel was observed in miR-133a-transfected OVCAR-3 cells, indicating that miR-133a regulates cell invasion in OVCAR-3 cells. miR, microRNA; NC, negative control. ^**^P<0.01 vs. the control group.

**Figure 4:**
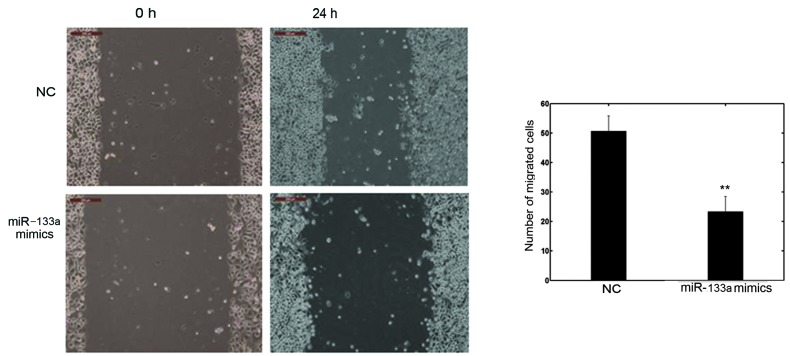
Scratch migration assay was performed to evaluate cell migration (magnification, ×100). OVCAR-3 cells were transfected with NC or miR-133a mimics and grown to confluence. A scratch was set through the dish and cells were cultured under standard conditions for 24 h. Following several washes, plates were photographed and the cells having migrated from the wound edge were counted. The number of migrated cells transfected with miR-133a mimics was significantly lower than with the NC, indicating that miR-133a affects OVCAR-3 cell migration. miR, microRNA; NC, negative control. ^**^P<0.01 vs. the control group.

The cell invasion and scratch migration assays were used to confirm the effects of miR-133a on cell invasion and migration, respectively. Following transfection with miR-133a mimics or NC in OVCAR-3, a significant downregulation of invasion into Matrigel was observed in miR-133a-transfected OVCAR-3 cells (Fig. 3). Furthermore, the number of migrated cells transfected with miR-133a mimics was significantly lower than with the NC, as shown in Fig. 4. These observations confirm the function of miR-133a in the invasion and metastasis of OVCAR-3 cells.

## Discussion

It is well known that ovarian cancer is a common gynecological malignancy and a leading cause of cancer mortality among females worldwide. Although progress has been made in ovarian cancer diagnosis and treatment, there are still numerous unexplored areas, and patients with metastasis or recurrent diseases continue to have a poor prognosis. miRNAs are important regulatory factors and are involved in a number of biological processes, including cell cycle regulation, cell growth, apoptosis, cell differentiation and stress response (24). Notably, miRNAs may have a modulatory role in oncogenic and tumor suppressor pathways (25). Investigation of miRNA activity in the human body may further develop the new and promising therapies for the treatment and management of human malignancies, including EOC.

The present study has demonstrated for the first time that miR-133a is downregulated in the OVCAR-3 ovarian cancer cell line and primary tumor samples. In addition, miR-133a was found to reduce OVCAR-3 cell viability, promote cell apoptosis and affect cell invasion and migration. Furthermore, the expression levels of miR-133a were markedly associated with clinical and pathological features, including tumor stage and grade, and lymph node metastasis. These findings suggest that miR-133a may be a useful target for therapeutic intervention and a biomarker for the prediction of EOC progression and prognosis.

Previous studies have illustrated that miR-133a plays a suppressive role in tumors. For example, ectopic miR-133a has been reported to suppress cell growth in lung cancer (26), maxillary sinus squamous cell carcinoma (27), tongue cancer (18), esophageal cancer (16), prostate cancer (28), bladder cancer (29) and renal cell carcinoma (19). Additionally, miR-133a was found to induce apoptosis in maxillary sinus squamous cell carcinoma (27), tongue cancer (18), bladder cancer (29) and renal cell carcinoma (19). miR-133a may also inhibit cell migration and invasion activities in esophageal cancer (16), prostate cancer (28), bladder cancer (29) and renal cell carcinoma (19). Furthermore, Wu *et al* revealed that loss of expression of miR-133a was markedly associated with tumor lymph node metastasis, advanced clinical stages and shortened relapse-free survival in patients with breast cancer (30). Collectively, these results suggest that miR-133a may be of vital importance in tumor initiation and in the development and progression of malignancy.

Several targets of miR-133a have recently been identified, including fascin homologue 1 (31), transgelin 2 (19,27), purine nucleoside phosphorylase (27), actin-related protein 2/3 complex subunit 5 (26), glutathione *S*-transferase π 1 (26), caveolin-1 (14), LASP1 (32) and pyruvate kinase M2 isoform (18), using qPCR, western blotting, reporter assays and bioinformatic prediction programs. However, the molecular genetic basis of carcinogenesis and cancer progression remains unclear. miRNAs may have a functional role, according to a combinatorial circuit model, in which a single miRNA may be used as the target of multiple mRNAs, and several coexpressed miRNAs may target a single mRNA. Studies are likely to remain far from unveiling all miR-133a targets, and the roles of some of the potential targets in EOC carcinogenesis and progression are poorly understood. Therefore, future research is required to identify the targetome and the exhaustive roles of miR-133a in ovarian cancer, based on this assumption.

In conclusion, the present study has demonstrated that miR-133a is downregulated in epithelial ovarian cancer and that decreased miR-133a expression is associated with advanced clinical stage, poor histological differentiation and lymph node metastasis. In addition, miR-133a plays a critical role in the cell viability, apoptosis, invasion and migration of ovarian cancer OVCAR-3 cells. These findings suggest that miR-133a may be a useful biomarker for the prediction of ovarian cancer progression and a potential promising target for gene therapy.

## Figures and Tables

**Table I:** Relationship between miR-133a expression level and clinicopathological features.

Factors	Patients, n	miR-133a (average fold-change ± standard deviation)	P-value
FIGO stage			
I, II	20	0.59±0.26	<0.001
III, IV	50	0.16±0.10	
Grade			
G1, G2	28	0.45±0.30	0.008
G3	42	0.21±0.17	
Lymph node			
Negative	46	0.51±0.34	0.003
Positive	24	0.18±0.15	
Histotype			
Serous	38	0.38±0.14	0.388
Non-serous	32	0.29±0.19	

miR, microRNA; FIGO, Federation of Gynecology and Obstetrics.
